# Supplementary figures and images for: Transcriptomic Signatures Predict Regulators of Drug Synergy and Clinical Regimen Efficacy against Tuberculosis
Source: mBio. 2019 Nov 12;10(6):e02627-19. doi: 10.1128/mBio.02627-19 (PMC6851285; doi:10.1128/mBio.02627-19)

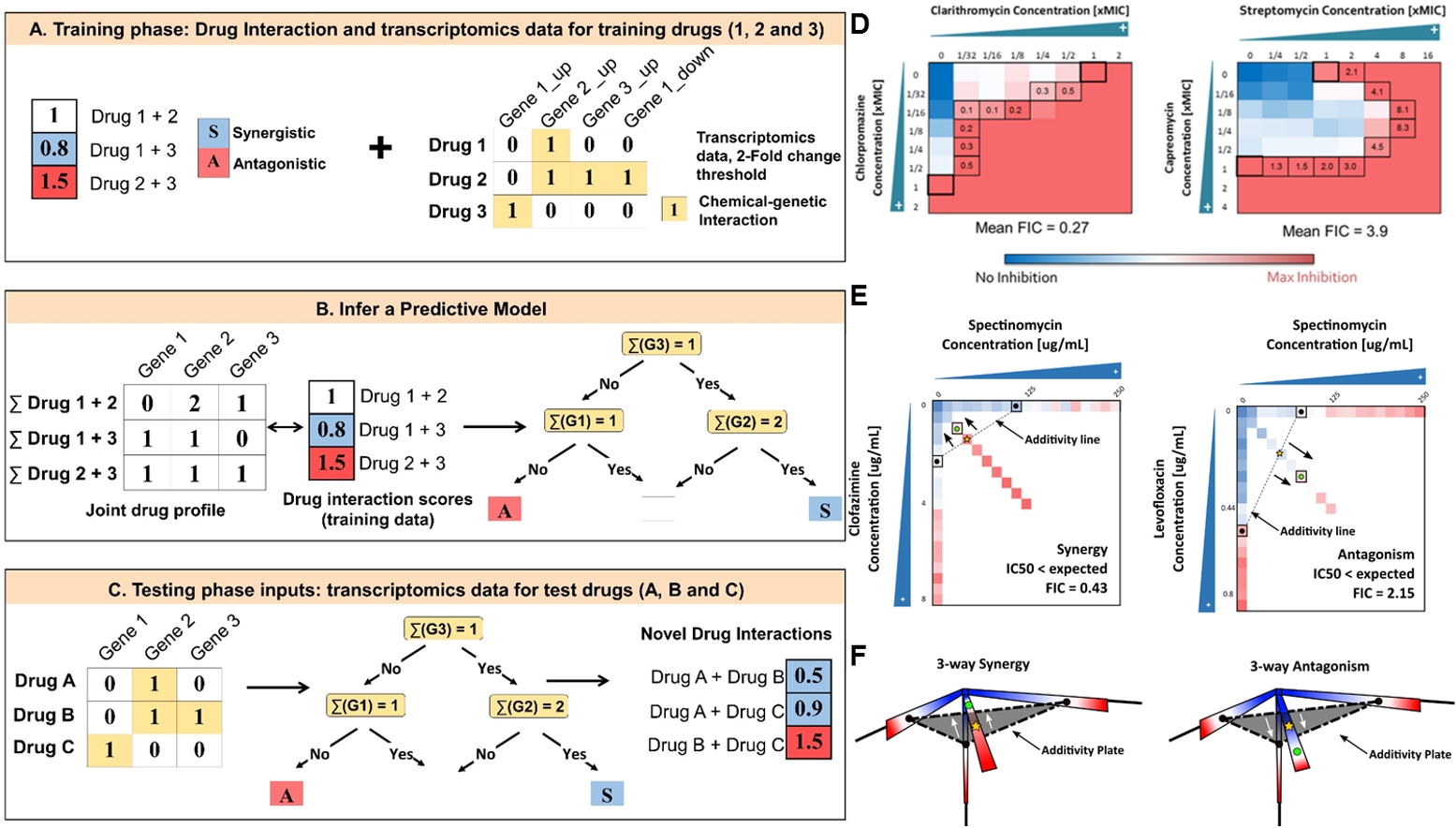

Supplement: FIG S1 [file mBio.02627-19-sf001.jpg]

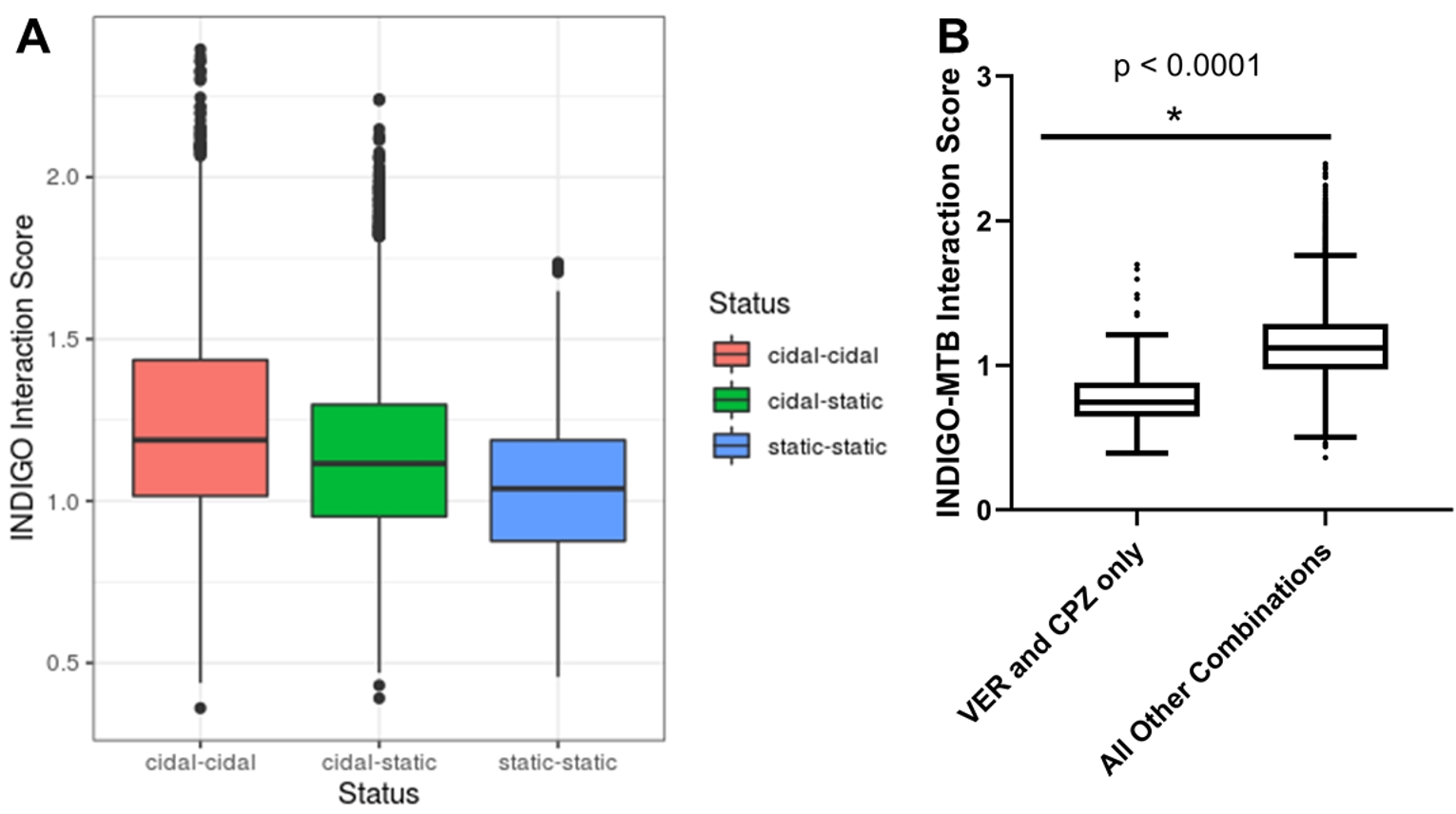

Supplement: FIG S2 [file mBio.02627-19-sf002.jpg]

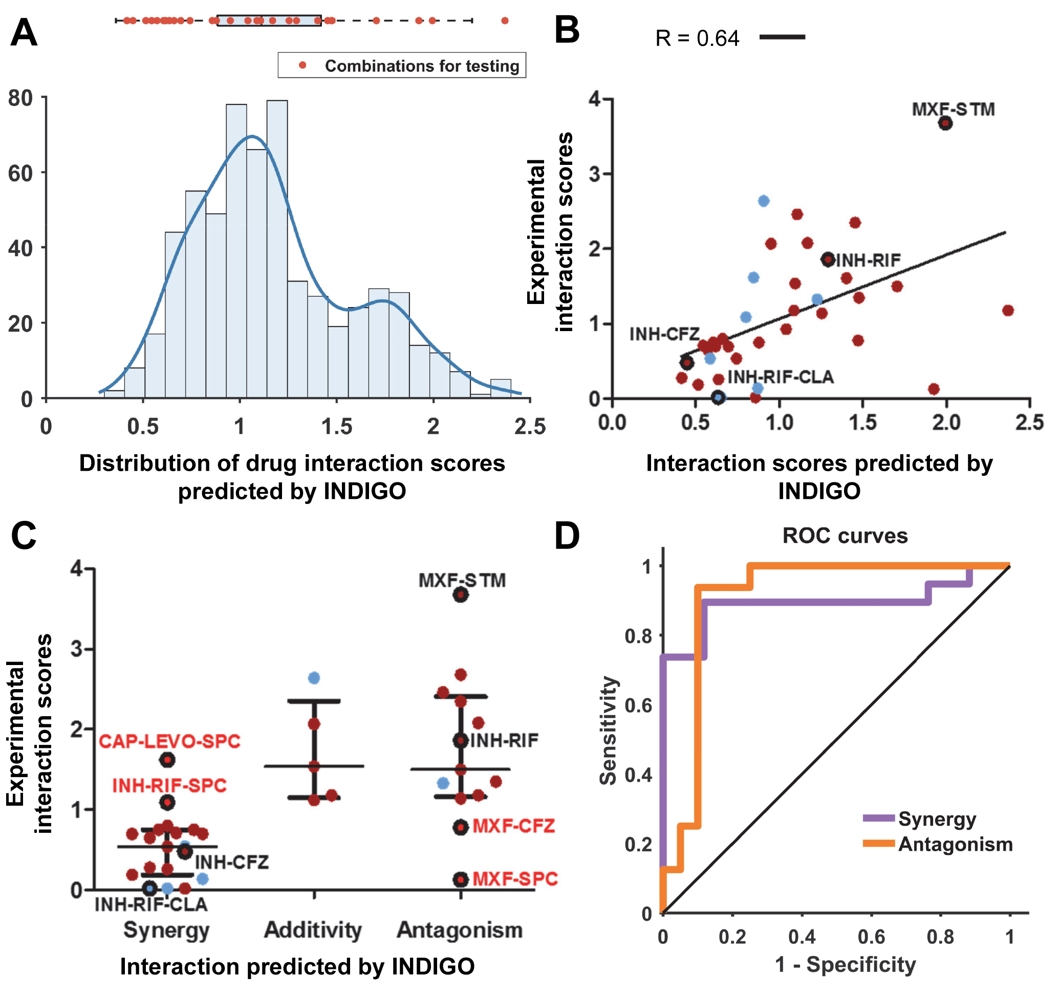

Supplement: FIG S3 [file mBio.02627-19-sf003.jpg]

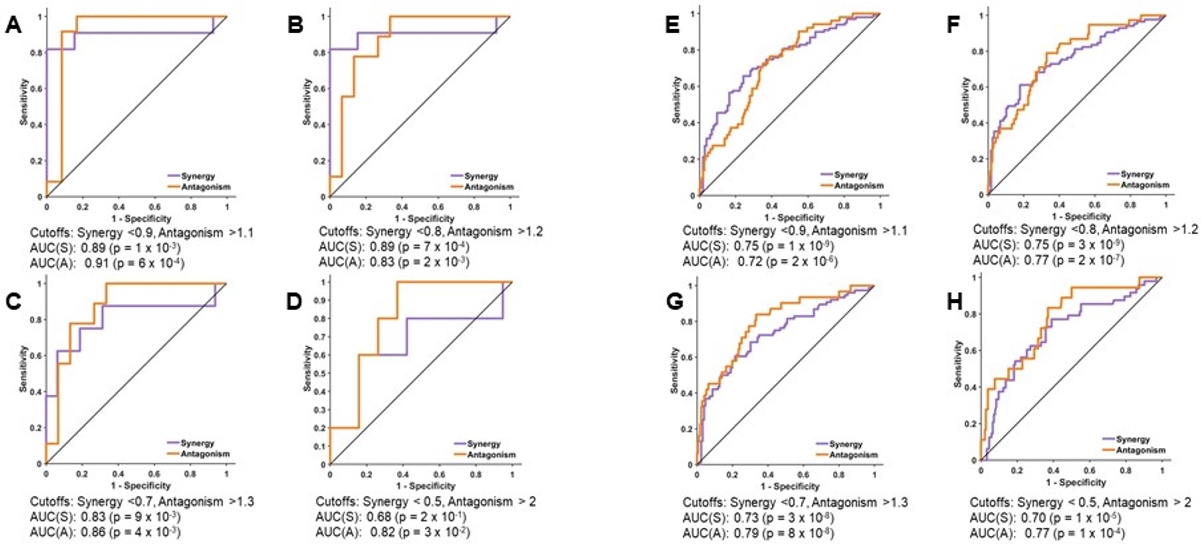

Supplement: FIG S4 [file mBio.02627-19-sf004.jpg]

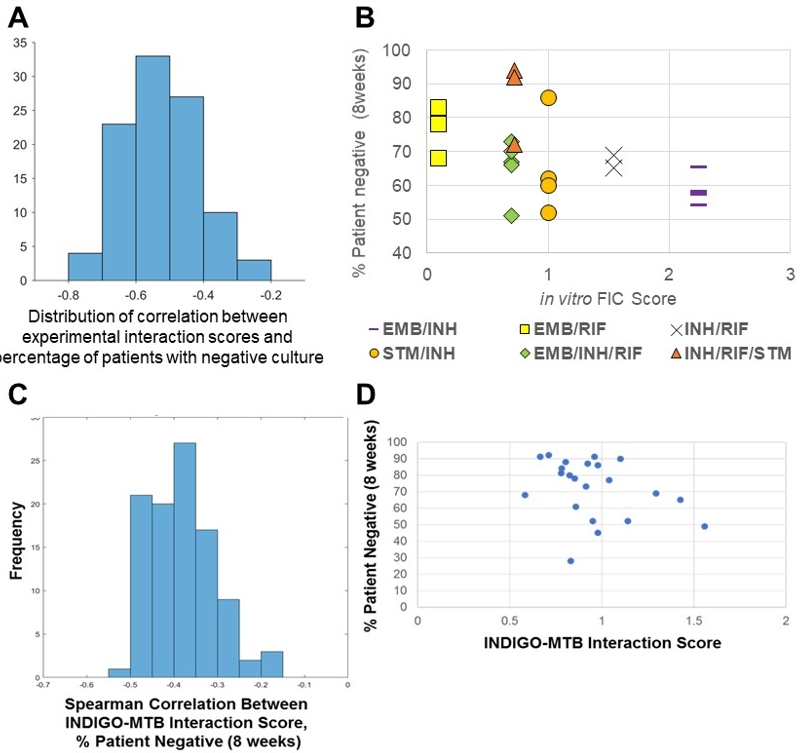

Supplement: FIG S5 [file mBio.02627-19-sf005.jpg]

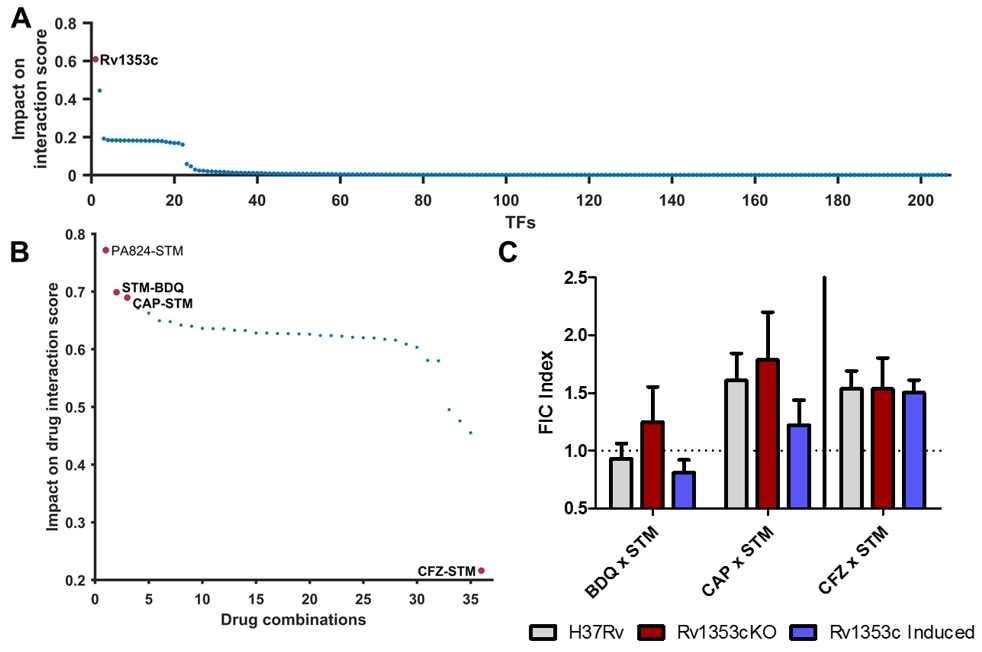

Supplement: FIG S6 [file mBio.02627-19-sf006.jpg]

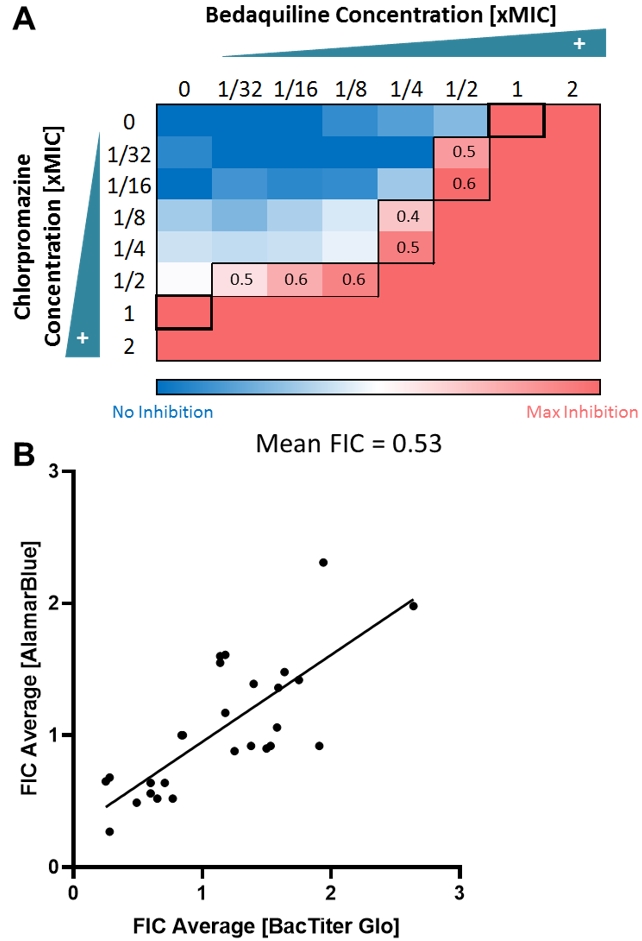

Supplement: FIG S7 [file mBio.02627-19-sf007.jpg]
